# Toripalimab plus chemotherapy as first-line treatment in extensive-stage small cell lung cancer: a health economic evaluation in China

**DOI:** 10.3389/fpubh.2025.1690043

**Published:** 2025-12-04

**Authors:** Jiaming Zhu, Zhengxiong Li, Wen Liu

**Affiliations:** 1 Fourth Clinical Medical College of Zhejiang Chinese Medical University, Affiliated Hangzhou First People’s Hospital, Hangzhou, China; 2 School of Medical Informatics and Engineering, Xuzhou Medical University, Xuzhou, China; 3 School of Humanities and Management, Zhejiang Chinese Medical University, Hangzhou, China

**Keywords:** health economic evaluation, extensive-stage small cell lung cancer, PD-1, drug-wastage, biomarkers

## Abstract

**Objective:**

To assess the cost-effectiveness of toripalimab plus etoposide and platinum (EP)-based chemotherapy as a first-line treatment for extensive-stage small cell lung cancer (ES-SCLC) from a Chinese healthcare system perspective, and to explore the impact of factors such as biomarker stratification, drug wastage, and drug donation on cost-effectiveness.

**Methods:**

A partitioned survival model was conducted to simulate the disease progression in ES-SCLC patients. Model parameters were derived from the EXTENTORCH clinical trial, public databases, and published literature. Key outcomes included total cost, quality-adjusted life years (QALYs), incremental cost-effectiveness ratio (ICER), and incremental net monetary benefit (INMB). Drug wastage was quantified based on body surface area, and patients were stratified according to biomarkers. Sensitivity analysis and scenario analysis were conducted to assess model stability.

**Results:**

Over a 10-year simulation period, the total cost of toripalimab plus EP was $28,551.37, with a QALYs of 0.75; the total cost of EP was $24,678.81, with a QALYs of 0.55. The ICER was $20,034.74/QALY, below China willingness-to-pay threshold of 2–3 times GDP. The sensitivity analysis demonstrated the stability of the conclusions and indicated that when the WTP is set at 2 times the GDP, toripalimab has an 89% probability of being cost-effective. The A11+/B62– genotype and the intratumor heterogeneity (ITH) low population achieve better efficacy and a lower ICER compared to the overall population. While drug wastage increases the treatment cost, it does not alter the conclusions.

**Conclusion:**

Compared to EP alone, toripalimab plus EP is cost-effective as first-line treatment in Chinese ES-SCLC patients, particularly when considering genetic stratification and donation policies. The study results provide important reference for China medical insurance policy-making and clinical practice.

## 1 Introduction

Lung cancer is the most prevalent and deadliest malignancy worldwide, accounting for 1/8 of global new cancer cases and 20% of cancer-related deaths in 2022 (1). Small cell lung cancer (SCLC), representing 15% of cases, is primarily associated with smoking and constitutes an extremely aggressive neuroendocrine carcinoma. Approximately 70% of SCLC patients present with distant metastases at diagnosis, resulting in dismal prognosis with a mere 17.6% three-year survival rate (2). In China, the disease burden of ES-SCLC aligns with global patterns but exhibits significant regional disparities, demonstrating notably higher incidence and mortality rates in high tobacco-consumption areas such as rural China (3). The clinical burden of SCLC is particularly severe. From a treatment cost perspective, SCLC patients demonstrate significantly higher chemotherapy utilization rates compared to non-small cell lung cancer (NSCLC), with chemotherapy and related expenses constituting 63% of total medical expenditures, representing the primary driver of direct economic burden (4). Regarding survival outcomes, SCLC prognosis is exceptionally poor, particularly for ES-SCLC patients, whose median survival typically falls below 1 year. This leads to substantial indirect costs associated with premature mortality, imposing severe socioeconomic impacts with average losses reaching $99,000 per patient (5). From a disease burden standpoint, the GBD 2019 study confirmed that 98.8% of lung cancer disability-adjusted life years stem from years of life lost rather than years lived with disability (6). As the most aggressive and rapidly progressing lung cancer subtype, SCLC serves as a key contributor to this phenomenon. Consequently, SCLC presents an urgent public health challenge both clinically and economically.

Due to its high aggressiveness and significant intra- and/or intertumoral heterogeneity, SCLC presents a greater therapeutic challenge than NSCLC, with limited breakthroughs in its treatment in recent years. Unlike limited-stage SCLC, ES-SCLC patients lose the opportunity for surgical intervention. First-line treatment is etoposide and platinum (EP), supplemented by immune checkpoint inhibitors (ICIs). Most immunotherapies target programmed death 1 (PD-1), with FDA-approved inhibitors including durvalumab and atezolizumab, both of which demonstrated modest survival benefits of only approximately 2 months in phase III clinical trials (7, 8). Despite this, up to 60% of patients still relapse within 3 months (2). Subsequently, the CheckMate-451 and KEYNOTE-158 trials revealed that nivolumab and pembrolizumab, PD-1 inhibitors that have shown positive clinical outcomes in other tumor types, did not significantly improve overall survival (OS) in ES-SCLC (7, 8). Therefore, the future of PD-1 inhibitors in the treatment of SCLC remains highly uncertain. Toripalimab is a novel, fully humanized IgG4 monoclonal antibody targeting PD-1. Structurally, it features an FG loop binding region, which confers a higher binding affinity to PD-1 compared with nivolumab and pembrolizumab, thereby enhancing immune activation while potentially reducing off-target adverse effects (9). Since its initial approval in China in 2018 for second-line treatment of melanoma, toripalimab has become the first anti-PD-1 monoclonal antibody licensed by the National Medical Products Administration (NMPA). Its indications have since expanded to include nasopharyngeal carcinoma, urothelial carcinoma, and NSCLC, demonstrating preliminary clinical efficacy and manageable toxicity profiles (10). Internationally, toripalimab was approved by the FDA in 2023 for nasopharyngeal carcinoma, and later by the EMA for the treatment of nasopharyngeal and esophageal squamous cell carcinomas. The EXTENTORCH phase III clinical trial conducted in China evaluated the survival benefits of adding toripalimab to EP in ES-SCLC. The results showed that the toripalimab group achieved a median OS of 14.6 months, compared to 13.3 months in the control group, representing a significant survival advantage and a 20% reduction in the risk of death. After excluding the confounding impact of second-line PD-1/PD-L1 inhibitor use on OS, the toripalimab group demonstrated a more pronounced clinical benefit, with median OS extended by more than 3 months (14.6 vs. 11.5 months). Additionally, the trial performed whole-exome sequencing on tumor biopsy samples and matched peripheral blood mononuclear cells, followed by exploratory biomarker analysis. The findings suggested that patients with low intratumor heterogeneity (ITH) (defined by MATH score <29), human leukocyte antigen (HLA)-A11 + HLA-B62-haplotypes, wild-type genes involved in focal adhesion/integrin signaling, CTNNA2 and SCN4A mutations, or wild-type KMT2D and COL4A4 were more likely to benefit from toripalimab. These results support a shift from a one-size-fits-all approach to precision immunotherapy guided by ITH scores and HLA-based genetic profiling, helping avoid unnecessary toxicity and economic burden associated with ineffective treatments (11).

As highlighted by Rajangom et al., drug wastage is most commonly associated with intravenously administered medications and may negatively impact cost-effectiveness while increasing patients’ financial burden. Strategies such as drug sharing and dose rounding can substantially reduce waste. For instance, in the United States, the median value of drug waste is $122.91 per patient per month, and mitigation measures could reduce wastage by up to 70% (12). During toripalimab plus EP, cisplatin and etoposide are administered based on body surface area (BSA), which often leads to mismatches between the required dose and the volume per vial, resulting in wastage. In addition, certain agents used in second-line treatment also contribute to drug waste.

The strong clinical performance of the EXTENTORCH trial has promoted toripalimab as a first-line treatment option for ES-SCLC and led to its recommendation as a category 1A treatment in the Chinese Society of Clinical Oncology (CSCO) guidelines (13). However, immune checkpoint inhibitors are associated with relatively high additional costs, requiring healthcare payers to carefully assess their cost-effectiveness. We aim to assess the economic value of adding toripalimab to standard EP chemotherapy versus EP monotherapy for initial treatment of ES-SCLC from the perspective of the Chinese healthcare system, based on data from the EXTENTORCH trial. It further explores the impact of precision medicine guided by genetic testing and drug wastage on cost-effectiveness, with the goal of providing scientific evidence for policymakers and pharmaceutical companies to support rational drug pricing and production strategies.

## 2 Methods

Implementation of the Consolidated Health Economic Evaluation Reporting Standards (CHEERS) 2022 guidelines governed this investigation’s reporting quality (14). Item-by-item validation against the 28-criterion checklist is systematically presented in Supplementary Table S1.

### 2.1 Target patient cohort and therapy

This study included patients diagnosed with ES-SCLC confirmed by histology or cytology. Eligible participants were required to have an Eastern Cooperative Oncology Group performance state (ECOG PS) score of 0 or 1 and at least one measurable lesion as defined by the Response Evaluation Criteria in Solid Tumors (RECIST) version 1.1. All enrolled patients had not received any prior systemic antitumor therapy for ES-SCLC. Patients were randomly assigned in a 1:1 ratio to either the experimental group or the control group. Each treatment cycle lasted 21 days, during which patients received an intravenous infusion of toripalimab 240 mg or matching placebo on day 1 of each cycle. Both groups concurrently received etoposide 100 mg/m^2^ on days 1–3 and EP agent (assumed to be cisplatin 75 mg/m^2^) on day 1, for a total of six cycles. Following chemotherapy completion, participants maintained toripalimab or placebo administration as monotherapy until either disease progression or unacceptable toxicity occurred. Routine follow-up was conducted in accordance with the CSCO guidelines for the diagnosis and treatment of SCLC (13). Upon disease progression, patients were allowed to receive subsequent antitumor therapies, with regimens and treatment proportions based on the data reported in the EXTENTORCH trial (11) (Supplementary Table S2).

### 2.2 Model construction

**Figure 1:**
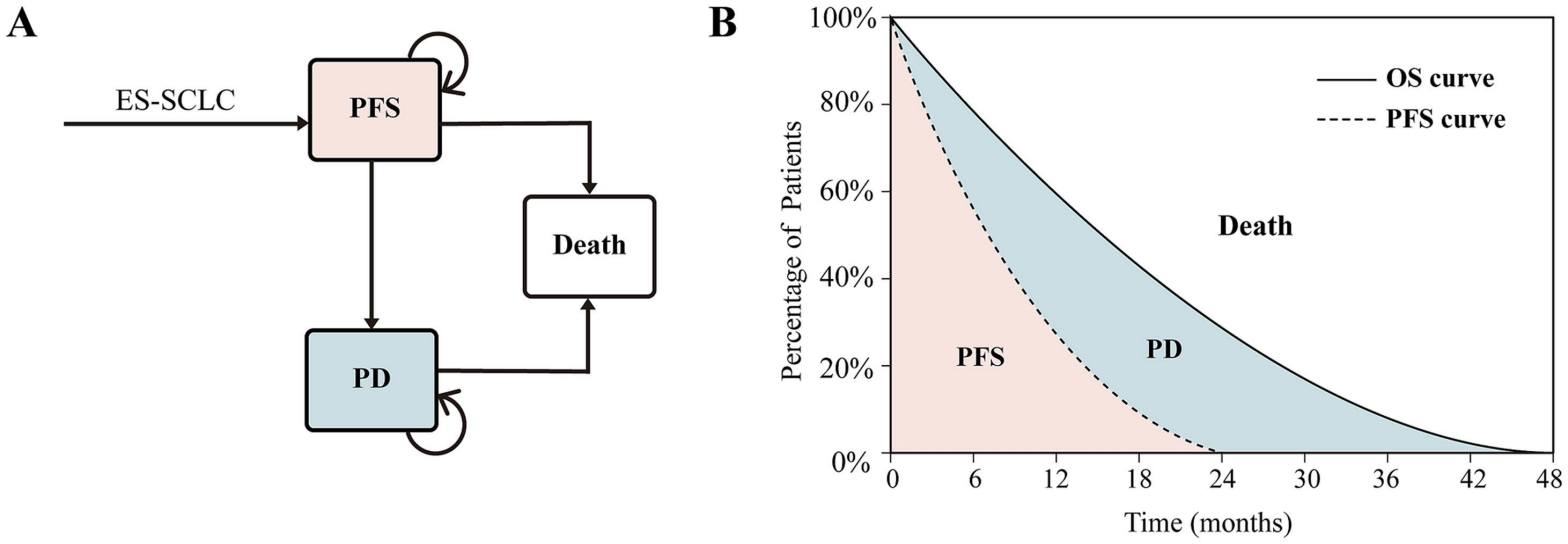
Structure of the partitioned survival model: specification of health state transition (**A**) and proportion of patients in different health states (**B**). ES-SCLC, extensive-stage small cell lung cancer; PD, progressive disease; PFS, progression-free survival; OS, overall survival.

A partitioned survival model was constructed using TreeAge Pro 2022 to simulate the disease progression of patients with ES-SCLC. The model included three mutually exclusive health states: progression-free survival (PFS), progressed disease (PD), and death. A unidirectional transition structure was applied, allowing patients to move only from PFS to PD or death, which reflects the natural history of disease progression. The proportion of patients in the PFS state was calculated using the area under the PFS survival curve. The PD state proportion was derived by subtracting the area under the PFS curve from the area under the OS curve. The death state proportion was estimated as the remainder of the total population not in the PFS or PD states (Figure 1). A cycle length of 21 days was adopted, and a 10-year analytical horizon was established to guarantee 99% of the cohort would enter the death state, capturing the long-term outcomes of treatment. Model outputs included total cost, quality-adjusted life years (QALYs), incremental cost-effectiveness ratio (ICER), net monetary benefit (NMB)=QALYs×WTP−Costs, and $\text{INMB}=(E_{\text{toripalimab}}-E_{\text{placebo}})\times \mathrm{WTP}-(C_{\text{toripalimab}}-C_{\text{placebo}})$. WTP thresholds were defined according to the *2020 China Guidelines for Pharmacoeconomic Evaluations*, with 1 × gross domestic product (GDP) per capita considered low-WTP, 2 × GDP medium-WTP, and 3 × GDP high-WTP. The per capita GDP in China in 2024 was estimated at $13,445. A 5% annual discount rate was applied (15).

### 2.3 Clinical data

The OS and PFS Kaplan–Meier (KM) curves were obtained from the phase III EXTENTORCH clinical trial. Individual patient data (IPD) were reconstructed from the KM curves using GetData Graph Digitizer, and the IPD reconstruction was performed in R software (Version 4.3.2) (16). Following recommendations from the National Institute for Health and Care Excellence (NICE) and the Canadian Agency for Drugs and Technologies in Health (CADTH), seven standard parametric survival distributions, including Exponential, Weibull, Gompertz, Gamma, Generalized gamma, Log-normal, and Log-logistic were fitted and extrapolated to the reconstructed data (16). The optimal distribution was selected based on Akaike Information Criterion (AIC), Bayesian Information Criterion (BIC), and visual inspection (17). Detailed distributed parameter, reconstructed and extrapolated curves are provided in Supplementary Table S3 and Supplementary Figures S1, S2.

### 2.4 Cost and utility

The study adopted a healthcare system perspective, incorporating direct medical expenditures encompassing drug costs, drug management costs, best supportive care cost, end-of-life care costs, and the costs associated with managing severe adverse events (SAEs) (18, 19). Drug costs were sourced from www.yaozh.com (20), local fee standards, and published literature (21–23). Grade 3–4 adverse events (AEs) were defined as SAEs, and the study only considered the management costs of SAEs with an incidence rate greater than 5% in both the toripalimab and control groups. The dosages of EP and second-line chemotherapy drugs were calculated based on BSA and weight, with an assumed BSA of 1.72 m^2^ and weight of 65 kg for the Chinese population (24). The management costs of SAEs were derived from published literature, with the total cost calculated as the cost of a single AE management multiplied by the reported incidence rate of that AE in the clinical trial. These costs were included only once in the first cycle of the model (25, 26). For the cost-effectiveness analysis of gene-stratified subgroups, the additional cost of genetic testing was included. Since genetic testing occurs during the diagnostic phase of the disease, the cost was incorporated into the first cycle of the model and included only once. All costs were adjusted to 2024 US dollars based on the annual inflation rate using https://www.inflationtool.com, and all RMB costs were converted to US dollars using the 2024 annual average exchange rate of 7.1217 RMB per 1 USD.

Utility values were used to measure patient satisfaction with their health status and overall well-being. All utility values used in this study were sourced from a publication that used the time trade-off method to measure health-related quality of life in NSCLC patients in Taiwan (27). Here, the utility values for NSCLC were used as a substitute for those of SCLC patients, consistent with previous studies (21). The utility decrement caused by SAEs was determined by multiplying the relevant disutility by the reported incidence of the SAEs in the EXTENTORCH clinical trial (Table 1).

**Table 1:** Key model parameters.

Parameter	Estimate	SA range	Distribution	Reference
Survival curve parameters				
OS of toripalimab: log-logistic	scale = 15.132; shape = 2.195			Model fitting
OS of placebo: log-logistic	scale = 13.058; shape = 2.397			Model fitting
PFS of toripalimab: log-logistic	scale = 6.21; shape = 2.3			Model fitting
PFS of placebo: log-logistic	scale = 5.406; shape = 3.339			Model fitting
Cost of drug (2024 $)				
Toripalimab per 240 mg	268.61	214.89–322.33	Gamma	(20)
Etoposide per 100 mg	23.45	18.76–28.14	Gamma	(20)
Cisplatin per 10 mg	1.12	0.90–1.34	Gamma	(20)
Irinotecan per 100 mg	89.71	71.77–107.65	Gamma	(20)
Anlotinib per 12 mg	39.81	31.85–47.77	Gamma	(20)
Atezolizumab per 1,200 mg	4,605.64	3684.51–5526.77	Gamma	(20)
Sintilimab per 100 mg	151.60	121.28–181.92	Gamma	(20)
Bevacizumab per 100 mg	150.95	120.76–181.14	Gamma	(20)
price of drug injection per time	2.99	2.39–3.59	Gamma	(35)
Cost of BSC per cycle	334.94	267.95–401.93	Gamma	(21)
Cost of genetic test	491.46	393.16–589.75	Gamma	Estimate
Cost of follow up of patients per unit	257.61	206.09–309.13	Gamma	(21)
Cost of terminal care per patient	8,170.86	6536.69–9805.03	Gamma	(21)
Cost of managing AEs (grade>3) per event				
Decreased neutrophil count	115.01	92.01–138.01	Gamma	(22)
Decreased WBC count	115.01	92.01–138.01	Gamma	(22)
Anemia	138.75	111.00–166.50	Gamma	(22)
Decreased platelet count	1,505.92	1204.74–1807.10	Gamma	(22)
Hyponatraemia	0.29	0.23–0.35	Gamma	(23)
Body surface (m^2^)	1.72	1.38–2.06	Gamma	(24)
Patient weight (kg)	65.00	1.38–2.07	Gamma	(24)
Discount rate	0.05	0–0.08	Fix	(15)
Health state utilities (QALYs)				
Progressive disease	0.62	0.50–0.74	Beta	(27)
Progression-free survival	0.69	0.55–0.82	Beta	(27)
Disutility due to AEs (grade>3)				
Decreased neutrophil count	0.20	0.16–0.24	Beta	(36)
Decreased WBC count	0.20	0.16–0.24	Beta	(22)
Anemia	0.07	0.06–0.09	Beta	(37)
Decreased platelet count	0.11	0.09–0.13	Beta	(22)
Hyponatraemia	0.09	0.07–0.11	Beta	(23)

QALY, quality-adjusted life year; BSC, best supportive care; WBC, white blood cell.

### 2.5 Sensitivity analysis

Deterministic sensitivity analysis (DSA) was performed to evaluate how changes in individual input variables affect the ICER. The analysis involved varying each variable by ±20% from baseline, with changes in the discount rate within a range of 0 to 8% (15). Notably, we allowed the price of toripalimab to fluctuate between 80 and 100% of its baseline value. This consideration is supported by the following reasons: Chinese charitable organizations provide assistance for eligible toripalimab buyers, which lowers the cost of obtaining the drug. Additionally, other indications of toripalimab have been included in the National Reimbursement Drug List (NRDL) in China, and should ES-SCLC be included as well, the price of toripalimab would not exceed $268.61 per 240 mg. The final results will present the top 20 variables that have the greatest impact on ICER in the form of a tornado diagram. To test the stability of the model, probabilistic sensitivity analysis (PSA) was conducted using 5,000 Monte Carlo simulations, introducing random fluctuations within a specified range for all input variables. According to ISPOR guidelines, variables related to BSA, weight, drug costs, and SAEs management costs were assigned a Gamma distribution, while utility values, SAEs incidence rates, and proportions of patients receiving different second-line treatments followed a Beta distribution, with a 10% baseline value used as the standard error range. The PSA results were presented using incremental cost-effectiveness scatter plots and cost-effectiveness acceptability curves.

### 2.6 Subgroup analysis

In the subgroup analysis, we stratified the population based on ITH and HLA-A11/B62 haplotype genotype. The analysis was conducted using the same method of base-case analysis to evaluate the cost-effectiveness of toripalimab in combination with EP compared to EP alone, followed by PSA. Survival analysis parameters, reconstructed curves, extrapolated plots are provided in Supplementary Tables S4–S6 and Supplementary Figures S3–S6.

### 2.7 Scenario analysis

#### 2.7.1 Drug wastage

In this treatment regimen, the dosage of some medications is calculated based on weight or BSA. Using a standard patient with a weight of 65 kg and a BSA of 1.72 m^2^, we calculated the difference between the theoretical drug dosage and the actual drug vial specifications. Once a drug vial is opened, it is used for only one patient, and the remaining drug is considered wasted. Based on this assumption, we quantified the changes in cost-effectiveness for first-line treatments (cisplatin and etoposide) and second-line treatments (irinotecan and bevacizumab) due to drug wastage.

#### 2.7.2 Patient assistance program

The Beijing Bethune Charitable Foundation launched a toripalimab donation program, providing free medication assistance to eligible patients. Under the assistance program, patients who have used toripalimab for four cycles on their own are eligible for an additional four cycles of the drug, with multiple applications allowed. Additionally, patients identified as low-income households can receive toripalimab free of charge. Our study analyzed the changes in cost-effectiveness due to the provision of free medication.

#### 2.7.3 Modification of treatment strategy

The EXTENTORCH clinical trial provided two chemotherapy regimens. In the base-case scenario, we assumed cisplatin as the chemotherapy drug. Here, we analyzed the cost-effectiveness of replacing cisplatin with carboplatin as the first-line chemotherapy drug. Additionally, the clinical trial only specifies the type of drugs used for second-line antitumor treatment, with the specific drug selection left to the discretion of the physician. Therefore, in this study, we selected the drugs recommended by the CSCO guidelines for SCLC as 1A evidence in the base case (13). However, in reality, the drug options extend beyond those assumed in the base-case scenario. In this scenario analysis, we replaced the domestic PD-1 inhibitor Sintilimab with Roche’s Atezolizumab for subsequent treatment to simulate the increased cost of second-line therapy. Furthermore, we simulated a scenario in which, after disease progression in the first-line treatment, no subsequent antitumor therapy is administered, and all patients receive best supportive care, thereby excluding the impact of second-line treatment costs on the decision-making process for the first-line treatment strategy.

## 3 Result

### 3.1 Base-case results

The base-case results showed that over a 10-year time horizon, the total costs for toripalimab combined with EP and placebo plus EP were $28,551.37 and $24,678.81, respectively, with the addition of toripalimab resulting in an additional cost of $3,872.56. In contrast, toripalimab combined with chemotherapy and chemotherapy alone yielded cumulative survival benefits of 0.75 QALYs and 0.55 QALYs, respectively, with an incremental survival benefit of 0.19 QALYs. Under WTP thresholds of 1–3 times GDP, the ICER was $20,034.74/QALY, which lies between low and medium WTP thresholds (Table 2). Additionally, at medium to high WTP thresholds, the INMB was positive, indicating that toripalimab plus EP is cost-effective compared to EP (Supplementary Table S7).

**Table 2:** Summary of model outputs with or without drug wastage, including the base case and 4-subgroup.

Regimen	QALYs	Incremental QALYs	Cost Without drug wastage ($)	Cost With drug wastage ($)	ICER Without drug wastage ($/QALY)	ICER With drug wastage ($/QALY)
Base-case						
Toripalimab + chemotherapy	0.75	0.19	28,551.37	29,067.11	20,034.74	19,811.00
Placebo + chemotherapy	0.55		24,678.81	25,237.79		
A11 + B62-						
Toripalimab + chemotherapy	0.91	0.32	32,243.01	32,805.60	18,174.72	18,119.62
Placebo + chemotherapy	0.58		26,373.24	26,953.63		
A11-B62+						
Toripalimab + chemotherapy	0.64	0.08	27,788.67	28,276.37	20,006.83	18,785.26
Placebo + chemotherapy	0.57		26,241.99	26,824.13		
ITH high						
Toripalimab + chemotherapy	0.47		24,531.33	24,949.88		
Placebo + chemotherapy	0.47	0.01	24,541.81	25,069.73	1,382.43	15,811.89
ITH low						
Toripalimab + chemotherapy	1.25	0.66	39,031.49	39,764.56	18,757.75	18,969.52
Placebo + chemotherapy	0.59		26,601.40	27,194.14		

ITH, intratumor heterogeneity; QALY, quality-adjusted life year; ICER, incremental cost-effectiveness ratio.

### 3.2 Sensitivity analysis

**Figure 2:**
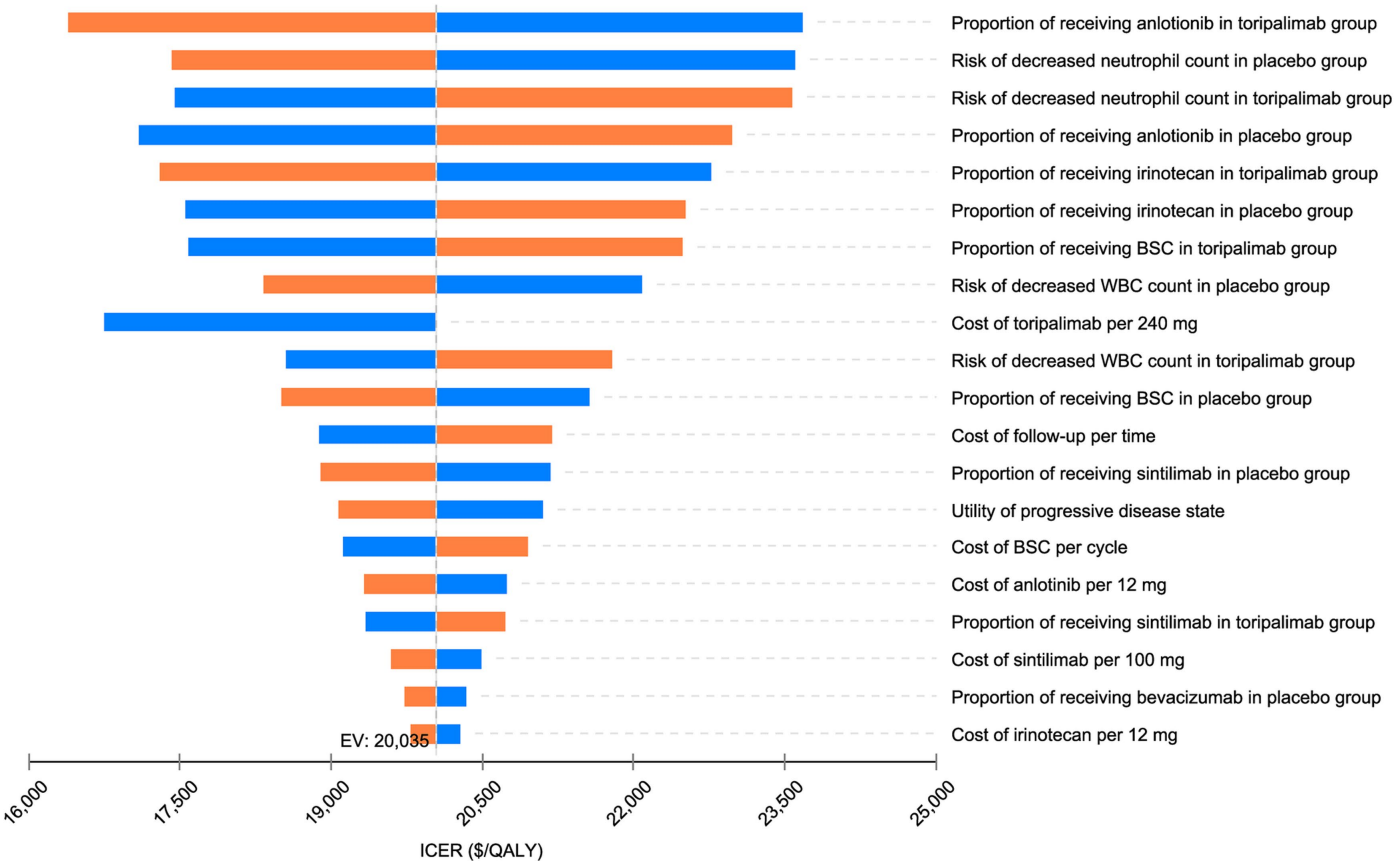
Deterministic sensitivity analysis: tornado diagram for incremental cost-effectiveness ratio for toripalimab plus EP vs. placebo plus EP. BSC, best supportive care; WBC, white blood cell; ICER, incremental cost-effectiveness ratio QALY, quality-adjusted life year, EV, expected value.

DSA revealed that the variables most influential on the ICER were the proportions of patients in the toripalimab and control groups receiving anlotinib as second-line treatment, the incidence of neutropenia in both the toripalimab and control groups, and the proportion of patients in the toripalimab group receiving irinotecan. Fluctuations within the specified ranges for all variables did not reverse the conclusions (Figure 2).

**Figure 3:**
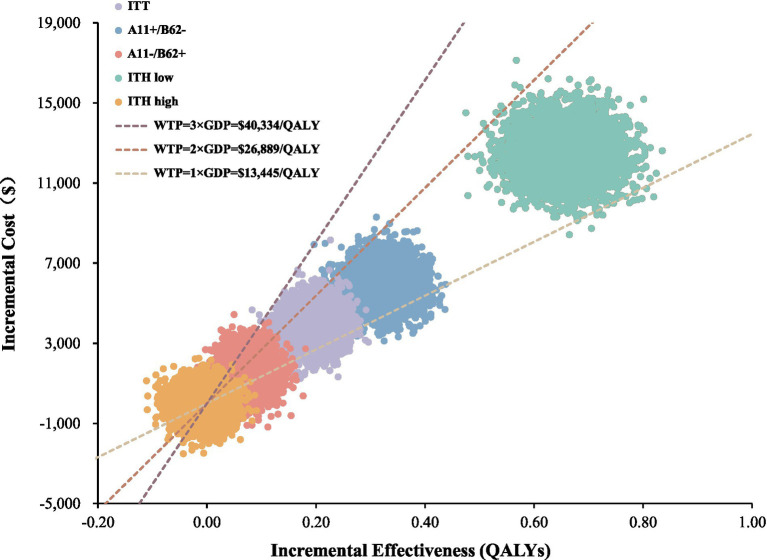
Incremental cost-effectiveness scatter plot for toripalimab plus EP vs. placebo plus EP in base-case and subgroup analysis. ITT, intention-to-treat; ITH, intratumor heterogeneity; WTP, willingness-to-pay; QALY, quality-adjusted life year.

**Figure 4:**
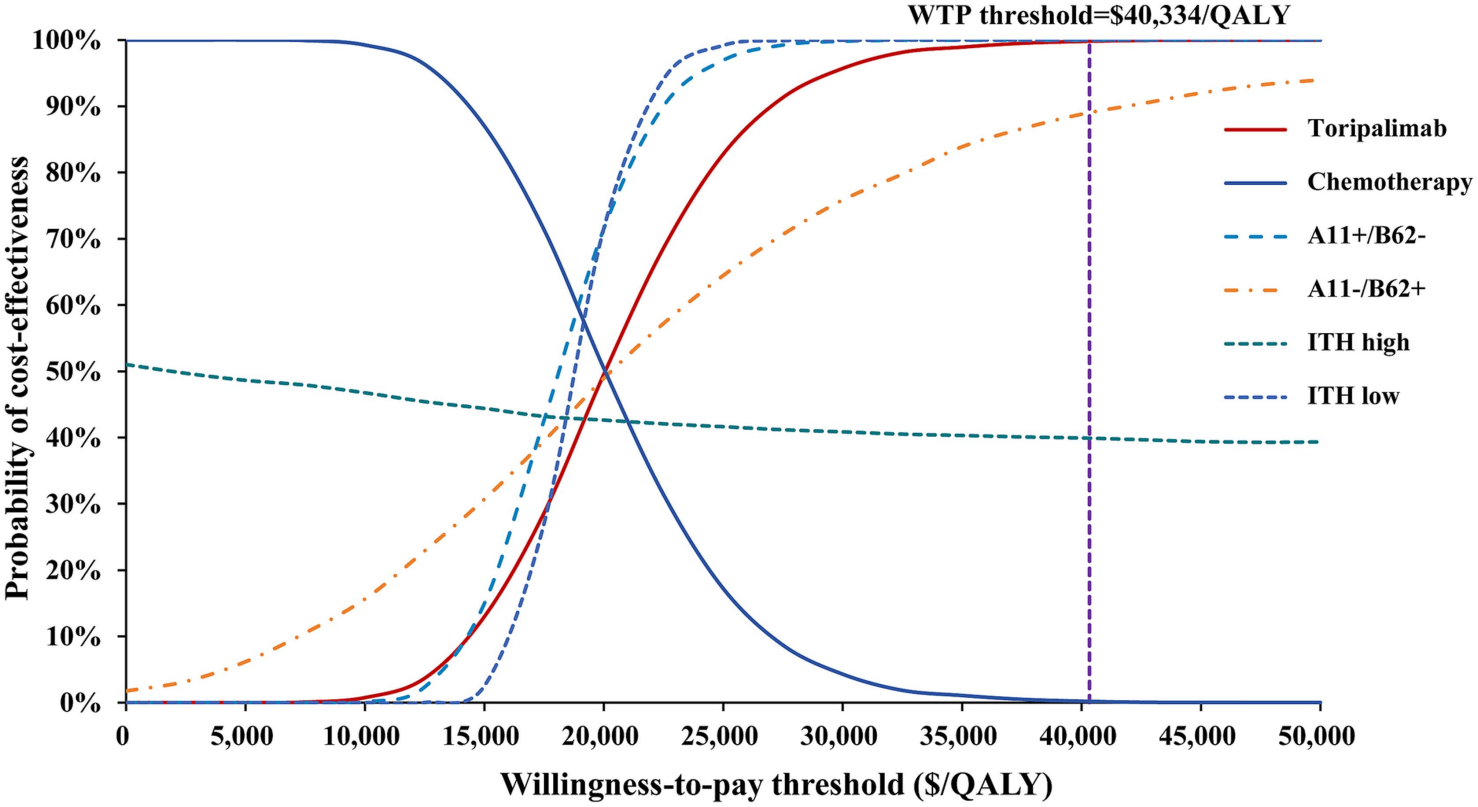
Cost-effectiveness acceptability curve for toripalimab plus EP vs. placebo plus EP in base-case and subgroup analysis. WTP, willingness-to-pay; QALY, quality-adjusted life year; ITH; intratumor heterogeneity.

The scatter plot showed that 83% of the points in the total intent-to-treat population fall between the low and medium WTP thresholds (Figure 3). The cost-effectiveness acceptability curve indicated that toripalimab becomes the more cost-effective option once the WTP exceeds $20,005.67/QALY. When the WTP is set at 3 × GDP, toripalimab has a 99.8% probability of being cost-effective, while at medium WTP and low WTP, the probabilities of toripalimab being cost-effective are 88.8 and 6.2%, respectively (Figure 4).

### 3.3 Subgroup analysis

In the subgroup analysis, the toripalimab-treated A11+/B62– population and A11–/B62 + population accumulated 0.91 QALYs and 0.64 QALYs, respectively, with ICERs of $18,174.72/QALY and $20,006.83/QALY indicating cost-effectiveness at medium WTP. In the incremental cost-effectiveness scatter plot, 93.5% of points in the A11+/B62– population and 43.6% of points in the A11–/B62 + population were located between the low and medium WTP thresholds. Additionally, one-quarter of the points for the A11–/B62 + group fell below the low WTP threshold. The cost-effectiveness acceptability curve showed that for the A11+/B62– population, when the WTP exceeds $18,109/QALY, the probability of toripalimab being cost-effective exceeds 50%. For the A11–/B62 + population, this threshold is $20,005.66/QALY.

In the high ITH population, the total cost and survival benefit in the control group were slightly higher than in the toripalimab group, resulting in an ICER of $1,382.43/QALY. This indicated that the addition of toripalimab did not lead to significant survival benefit but did not add extra cost burden, making the toripalimab regimen not cost-effective compared to chemotherapy. In the scatter plot, about half of the points showed higher costs for the toripalimab group compared to the chemotherapy group, while 40% of the points showed better clinical benefit for the toripalimab group. The cost-effectiveness acceptability curve showed a slow downward trend. When the WTP was below $1,129.35/QALY, toripalimab became the more cost-effective option, while at higher WTP, the chemotherapy group had a 60% probability of being cost-effective. For the low ITH population, with a total cost of $39,031.49 and 1.25 QALYs, the ICER was $18,757.75/QALY, demonstrating cost-effectiveness under the medium WTP (Table 2). The PSA showed that the toripalimab regimen had a 99.5% probability of being cost-effective under medium WTP. The scatter plot indicated that almost all points were located between the low and medium WTP thresholds. The cost-effectiveness acceptability curve showed that when the WTP exceeded $18,714.98/QALY, toripalimab was more cost-effective than chemotherapy (Figures 3, 4).

### 3.4 Scenario analysis

Regarding drug wastage, the treatment with the highest wastage in first-line therapy was etoposide, with a wastage of 84 mg ($19.70) per cycle. In second-line therapy, the treatment with the highest wastage is bevacizumab, with a wastage of 250 mg/cycle ($377.38/cycle), representing wastage rates of 16.3 and 2.6%, respectively. In the base-case scenario, the total cost increase due to drug wastage in the toripalimab group was $315.74, leading to a 34% reduction in NMB, whereas the total cost increase in the chemotherapy group was $558.30, resulting in a 19% decrease in NMB (Supplementary Table S8). The ICER decreased to $19,811.00/QALY. In other subgroups, the impact of drug wastage on ICER was most significant in the ITH high subgroup, where the ICER increased by 10 times after accounting for drug wastage. In both the base-case scenario and all subgroups, drug wastage did not alter the conclusions.

With the assistance of the Patient Assistance Program (PAP), the cost of toripalimab for patients can be reduced by half or even made completely free. In this scenario, the ICER showed a declining trend, falling below the low WTP threshold. The change in first-line chemotherapy drugs led to a decrease in ICER to $20,025.89/QALY, with no significant impact. The change in second-line treatment strategies indicated that as the cost of second-line therapy increased, the per-cycle drug cost in the PD state rose from $537.61 to $1,135.64, resulting in an ICER reduction to $-11,697.37/QALY, suggesting that toripalimab becomes the dominant treatment option compared to chemotherapy. If subsequent anticancer treatments are not adopted, the ICER for the toripalimab treatment group increases to $25,244.48/QALY.

## 4 Discussion

SCLC accounts for 15–20% of all lung cancers and is one of the most poorly differentiated and aggressive types. As a neuroendocrine tumor, SCLC secretes biologically active amines that can cause paraneoplastic syndromes, which complicate treatment and lead to a decreased survival rate, with a 5-year survival rate of less than 7% (20). The treatment of SCLC has long seen limited progress, primarily relying on chemotherapy (such as platinum-based regimens combined with etoposide) and radiotherapy. With the advancement of tumor immunology, immunotherapy particularly ICIs represent an innovative and effective therapeutic approach for various cancers. In recent years, the combination of immune checkpoint inhibitors and chemotherapy has become the standard first-line treatment for ES-SCLC, significantly improving survival rates for some patients, particularly the 3-year survival rate (2).

From the standpoint of China healthcare system, no prior study has analyzed the cost-effectiveness of toripalimab and chemotherapy in treating ES-SCLC. It also explores the potential impact of gene-based precision medicine and drug wastage on cost-effectiveness. The results indicate that, in contrast to traditional chemotherapy regimens, the cost per QALY gained with toripalimab plus EP is $8,178.52, which is below the threshold defined by China 2024 per capita GDP. Furthermore, based on sensitivity analyses, this study concludes that toripalimab combined with chemotherapy is a cost-effective treatment option. These findings hold clear practical value for clinical practice in China. Methodologically, the study design strictly aligns with first-line treatment pathways outlined in CSCO guidelines, while incorporating China-specific healthcare system factors, including immunotherapy accessibility (e.g., charitable assistance) and guideline-concordant post-progression management (second-line regimen selection and best supportive care integration). These considerations enhance alignment between the conclusions and the actual operation of China’s healthcare system, enabling direct evidence-based support for two key decision-making areas: first, providing economic evidence for healthcare insurance authorities to evaluate toripalimab’s reimbursement eligibility for ES-SCLC, supporting dynamic adjustments to the NRDL; second, assisting medical institutions (particularly tertiary hospitals with sufficient resources) in optimizing ES-SCLC clinical pathways by balancing efficacy and cost in individualized treatment planning. Notably, real-world deviations from ideal study conditions may exist, though they do not undermine the core conclusion. These include regional disparities in treatment accessibility due to uneven medical resource distribution (e.g., limited immunotherapy stock in primary hospitals), variations in guideline adherence across institutions (e.g., non-recommended second-line regimens adopted for clinical or economic reasons), and off-label drug use (e.g., off-indication or dosage adjustments), which may marginally impact cost-effectiveness estimates.

HLA-A and HLA-B genes are located on the same chromosome in close proximity and are often inherited as haplotypes in the population (28). The EXTENTORCH trial demonstrated that patients with the HLA-A11+/B62– haplotype receiving toripalimab combination therapy showed superior PFS and OS benefits. Among all patients receiving toripalimab treatment, the A11+/B62– subgroup had a median PFS extended by 1.5 months and OS extended by 6.5 months compared to the A11–/B62 + subgroup. In this study, the A11+/B62– population accumulated more QALYs under toripalimab treatment than the A11–/B62 + population, thereby providing better cost-effectiveness, which aligns with the findings from the clinical trial. In comparison to the survival benefits in the base-case population, the survival benefits accumulated with chemotherapy for both the A11+/B62– and A11–/B62 + groups were similar. However, after the addition of toripalimab, the accumulated utility value for the A11+/B62– group was significantly higher than that of the entire treatment population, suggesting that the A11+/B62– genotype could serve as a biomarker for better clinical outcomes and cost-effectiveness with toripalimab. In East Asia, the prevalence of HLA-A11 is relatively high, with 26.8% of the Chinese population carrying the A11 gene (29), compared to less than 5% in the White population (30, 31). This difference implies that the stratified treatment strategy based on the HLA-A11 allele, which showed value in guiding toripalimab treatment decisions in Chinese cohort, may have limited utility in clinical practice in European and American countries.

The level of ITH is a stronger differentiator for the expected cost-effectiveness and prognosis of toripalimab treatment across different populations. In the ITH high population, the survival benefits of toripalimab were nearly identical to those of chemotherapy, and even worse than in the base-case scenario. In contrast, the ITH low population receiving toripalimab showed significantly higher QALYs compared to both the ITH high and overall populations. However, due to the larger proportion of patients in the PFS group, a higher number of patients received toripalimab, leading to increased costs. The ICER for this group did not differ significantly from the base-case scenario, but the NMB and INMB showed significant differences. SCLC is characterized by high ITH, with tumor cells exhibiting varied sensitivity to different drugs, which results in the development of multiple resistances. When the MITH score is below 29, ITH is defined as low, and tumor cell clones are more homogeneous. This allows PD-1 inhibitors to effectively activate T cells (32, 33). This subgroup analysis suggests that ITH levels are an important reference for selecting patients for toripalimab treatment in ES-SCLC. However, since ITH scores depend on tumor tissue testing, which is more costly than peripheral blood-based HLA typing, its use in clinical practice is limited and may be difficult to implement. Therefore, when selecting ideal patient populations for toripalimab in real-world healthcare systems, the necessity of considering MITH scores should be evaluated cautiously. Nonetheless, the use of gene-based biomarker screening to guide high-affinity drug selection in ES-SCLC treatment is a valuable practice to enhance drug cost-effectiveness and promote precision medicine.

In one of our scenario analyses, drug wastage was shown to increase the costs of both first-line and second-line treatments by 5%, highlighting the importance of reducing drug wastage. Due to the greater impact of drug wastage on the costs in the chemotherapy group compared to the toripalimab group, the ICER decreased after accounting for drug wastage. In this context, the reduction in the NMB indicator more accurately reflects the economic impact of drug wastage. For example, in the case of etoposide, assuming a patient with a BSA of 1.72 m^2^, two vials of 100 mg etoposide would be required, resulting in a wastage rate of 14%. If the patients BSA exceeds 2 m^2^, at least three vials would need to be opened, potentially leading to even greater drug wastage. This underscores the need for optimizing or reducing the dosage per vial or implementing billing reforms (e.g., adopting billing based on actual drug consumption) to mitigate the economic toxicity associated with drug wastage. Given China large population and substantial patient base, the establishment of “Drug Days,” where unified drug administration and vial sharing are implemented, could be a beneficial strategy to reduce drug wastage (12). This practice aligns with China’s national initiatives. In December 2023, five national authorities including the National Health Commission and the State Administration for Market Regulation jointly issued a notice on the Implementation Plan for Conserving Pharmaceutical Resources and Curbing Drug Wastage. The plan puts forward specific requirements covering clinical drug use, pharmaceutical distribution, and other aspects, demonstrating a full-lifecycle approach to curbing drug wastage from the source to the end-user. In another scenario analysis, replacing cisplatin with carboplatin in first-line treatment did not significantly alter the ICER, as the treatment costs for both drugs were similar per cycle. However, in second-line treatment, a notable proportion of patients (13.9% in the toripalimab group and 25.1% in the placebo group) were retreated with PD-1/PD-L1 inhibitors. This resulted in a significant cost difference, which substantially affected the ICER. Nevertheless, this may not accurately reflect the economic viability of toripalimab as a first-line ES-SCLC treatment. Therefore, excluding the effects of second-line treatment provides a clearer evaluation of the cost-effectiveness of toripalimab.

A network meta-analysis indirectly compared the health economic evaluation of four ICI-based combination therapies as first-line treatments for ES-SCLC (23). The results indicated that, at a WTP threshold of three times the GDP in 2022, ICI combined with EP was not cost-effective compared to EP alone. The most cost-effective regimen was serplulimab combined with chemotherapy; however, the high drug cost driven by its price still made the clinical benefit unaffordable. Price simulations showed that serplulimab would need a price reduction of at least 86.7% to reach the cost-effectiveness threshold. In comparison, the cost per cycle of toripalimab in this study was only $271.60, while serplulimab, which has not been included in the national insurance program, had a cost of $784.64 per cycle. Since toripalimab was approved by the NMPA in 2018, it has undergone two price adjustments. After its inclusion in the national insurance program in 2020, the price dropped from $1010.99 to $268.48 per 240 mg, reflecting a 73% decrease. If the initial market price were applied, the ICER for toripalimab in ES-SCLC treatment would increase to $65,368.22/QALY, leading to conclusions similar to those for other newly launched ICIs. The primary advantage of toripalimab in treating ES-SCLC stems from the price advantage brought about by its inclusion in the national insurance program. It is worth noting that this price advantage may be more aligned with the context of China’s healthcare markey. This is because the pricing systems in global markets and medical insurance reimbursement policies vary significantly across different regions. For example, the prices of original PD-1 inhibitors in high-income countries like the United States and the European Union are typically much higher than toripalimab’s post-insurance price in China (34); in the absence of analogous price adjustment mechanisms, the ICER would tend to exceed local WTP thresholds in these areas. Prior economic evaluations further demonstrate that the economic profile of toripalimab varies by cancer type. When combined with chemotherapy, it proves cost-effective in triple-negative breast cancer and NSCLC but fails to meet cost-effectiveness thresholds in esophageal squamous cell carcinoma and renal cell carcinoma. This is because, in SCLC and triple-negative breast cancer, the incremental QALYs gain with toripalimab treatment compared to chemotherapy was large, suggesting that SCLC, NSCLC, and triple-negative breast cancer are high-value indications for toripalimab, whereas esophageal squamous cell carcinoma is a low-value indication. Toripalimab would need a 40% price reduction to achieve cost-effectiveness for esophageal squamous cell carcinoma (Table 3). Based on this fact, future pricing adjustments for toripalimab should consider moving away from the traditional national unified pricing model and instead adopt different pricing strategies for different indications, or implement a value-based pricing model based on individual efficacy. To our knowledge, previous studies have not considered drug wastage, a prevalent issue in China that has often been overlooked, but is of practical significance for the future Chinese market and drug production.

**Table 3:** Summary of published cost-effectiveness analysis on toripalimab.

Indications	Model and time horizon (year)	Cost of toripalimab per 240 mg (adjusted to 2024USD)	Incremental QALYs	ICER ($/QALY)	Conclusion	Reference
NSCLC	PSM, 10	347.50	0.57	32,237.00	Cost-effective	(38)
NSCLC	Markov, 30	260.86	0.64	9,445.00	Cost-effective	(39)
TNBC	PSM, 10	265.49	0.74	16,133.18	Cost-effective	(40)
RCC	PSM, 20	268.81	0.65	64,337.49	Not cost-effective	(41)
ESCC	PSM, 3	268.62	0.26	43,405.09	Requiring a price reduction of ≥40% to be cost-effective	(42)

NSCLC, non-small cell lung cancer; TNBC, triple-negative breast cancer; RCC, renal cell carcinoma; ESCC, esophageal squamous cell carcinoma; QALY, quality-adjusted life year; ICER, incremental cost-effectiveness ratio; PSM, Partitioned survival model.

## 5 Limitation

First, our analysis is heavily reliant on data from the single EXTENTORCH clinical trial, and the strict inclusion criteria of this single-trial cannot fully reflect the heterogeneity of patients in real-world settings. Due to the unavailability of IPD from the trial, we applied the Guyot algorithm to extract pseudo-IPD data from the published Kaplan–Meier survival curves (16). However, pseudo-IPD reconstruction may be inaccurate due to digitization errors of the curves or biases from the assumptions made (e.g., selection of survival models). Second, the analysis depends on extrapolating short-term trial data to long-term health outcomes. The extrapolation of pseudo-IPD data is limited by the follow-up duration of the EXTENTORCH trial. The median follow-up time in the toripalimab arm of the EXTENTORCH clinical trial was 13.7 months. This short-term data may not capture late-stage survival trends (e.g., potential long-term survival plateaus or late relapses) or long-term quality of life impacts, which are important for lifetime QALYs estimation. To validate these findings, future work could consider external validation, such as incorporating real-world long-term survival data from SCLC registries (e.g., the Chinese National Cancer Center SCLC Registry) to anchor extrapolations. Third, the incidence of SCLC is generally associated with smoking. Although the EXTENTORCH trial explored common gene mutations related to SCLC in the smoking population, such as CTNNA2/SCN4A mutations, KMT2D/COL4A4 mutations, and mutations related to focal adhesion/integrin signaling pathways, and identified relationships with clinical benefits, the trial did not fully report the number of individuals with risk events. Therefore, we are unable to assess the cost-effectiveness of toripalimab for this subgroup of patients. Finally, in the analysis, we had to make assumptions about the treatment regimens and follow-up for patients after progression. These assumptions were based on expert guidance and may not accurately reflect real-world drug preferences.

## 6 Conclusion

Based on our model, the ICER for toripalimab combined with chemotherapy is $20,034.74/QALY, making it a more cost-effective first-line treatment strategy for ES-SCLC compared to chemotherapy at medium and high WTP thresholds. Precision treatment based on the A11/B62 biomarker and ITH scores further enhances its cost-effectiveness. Our study provides evidence-based support for clinicians in treatment decision-making and for policymakers. Since the data are derived from a Chinese cohort, this study is particularly relevant to East Asia, while additional robust evidence is needed to support its applicability in Europe and the Americas.

## Data Availability

The original contributions presented in the study are included in the article/Supplementary material, further inquiries can be directed to the corresponding author.
