# Supplementary material for: Toripalimab plus chemotherapy as first-line treatment in extensive-stage small cell lung cancer: a health economic evaluation in China
Source: Front Public Health. 2025 Dec 4;13:1690043. doi: 10.3389/fpubh.2025.1690043 (PMC12711754; doi:10.3389/fpubh.2025.1690043)
Supplement: Supplementary file 1 [file Data_Sheet_1.pdf]

Supplementary Material

Supplementary Table S1 CHEERS Checklist (2022).

Supplementary Table S2 The proportions of patients receiving subsequent anti-cancer therapy and the probabilities of adverse events listed in the Phase III EXTENTORCH clinical trial.

Supplementary Table S3. Summary of the statistical goodness-of-fit of Kaplan Meier survival curves in overall population**.**

Supplementary Table S4 Summary of the statistical goodness-of-fit of Kaplan Meier survival curves in HLA-A11/B62 subgroup.

Supplementary Table S5 Summary of the statistical goodness-of-fit of Kaplan Meier survival curves in ITH subgroup.

Supplementary Table S6 Optimal distribution and survival parameters for all subgroup.

Supplementary Table S7 Summary of INMB in base-case and subgroup analysis at WTP thresholds of 1-3 times per capita GDP.

Supplementary Table S8 Summary of NMB in base-case and subgroup analysis at WTP thresholds of 1-3 times per capita GDP.

Supplementary Figure S1 Reconstruction of Kaplan Meier survival curve in the intention-to-treat population.

Supplementary Figure S2 Fitting and extrapolation of Kaplan Meier survival curve in the intention-to-treat population.

Supplementary Figure S3 Reconstruction of Kaplan Meier survival curve in HLA-A11/B62 subgroup.

Supplementary Figure S4 Reconstruction of Kaplan Meier survival curve in ITH subgroup.

Supplementary Figure S5 Fitting and extrapolation of Kaplan Meier survival curve in HLA-A11/B62 subgroup.

Supplementary Figure S6 Fitting and extrapolation of Kaplan Meier survival curve in ITH subgroup.

Supplementary Table S1. CHEERS Checklist (2022)

Topic	No.	Item	Reported?
**Title and abstract**			
Title	1	Identify the study as an economic evaluation and specify the interventions being compared.	Yes
Abstract	2	Provide a structured summary that highlights context, key methods, results, and alternative analyses.	Yes
**Introduction**			
Background and objectives	3	Give the context for the study, the study question, and its practical relevance for decision making in policy or practice.	Yes
**Methods**			
Health economic analysis plan	4	Indicate whether a health economic analysis plan was developed and where available.	Not applicable
Study population	5	Describe characteristics of the study population (such as age range, demographics, socioeconomic, or clinical characteristics).	Yes
Setting and location	6	Provide relevant contextual information that may influence findings.	Yes
Comparators	7	Describe the interventions or strategies being compared and why chosen.	Yes
Perspective	8	State the perspective(s) adopted by the study and why chosen.	Yes
Time horizon	9	State the time horizon for the study and why appropriate.	Yes
Discount rate	10	Report the discount rate(s) and reason chosen.	Yes

Selection of outcomes	11	Describe what outcomes were used as the measure(s) of benefit(s) and harm(s).	Yes
Measurement of outcomes	12	Describe how outcomes used to capture benefit(s) and harm(s) were measured.	Yes
Valuation of outcomes	13	Describe the population and methods used to measure and value outcomes.	Yes
Measurement and valuation of resources and costs	14	Describe how costs were valued.	Yes
Currency, price date, and conversion	15	Report the dates of the estimated resource quantities and unit costs, plus the currency and year of conversion.	Yes
Rationale and description of model	16	If modelling is used, describe in detail and why used. Report if the model is publicly available and where it can be accessed.	Yes
Analytics and assumptions	17	Describe any methods for analysing or statistically transforming data, any extrapolation methods, and approaches for validating any model used.	Yes
Characterising heterogeneity	18	Describe any methods used for estimating how the results of the study vary for subgroups.	Yes
Characterising distributional effects	19	Describe how impacts are distributed across different individuals or adjustments made to reflect priority populations.	Yes
Characterising uncertainty	20	Describe methods to characterise any sources of uncertainty in the analysis.	Yes
Approach to engagement with patients and others affected by the study	21	Describe any approaches to engage patients or service recipients, the general public, communities, or stakeholders (such as clinicians or payers) in the design of the study.	Not applicable
**Results**			

Study parameters	22	Report all analytic inputs (such as values, ranges, references) including uncertainty or distributional assumptions.	Yes
Summary of main results	23	Report the mean values for the main categories of costs and outcomes of interest and summarise them in the most appropriate overall measure.	Yes
Effect of uncertainty	24	Describe how uncertainty about analytic judgments, inputs, or projections affect findings. Report the effect of choice of discount rate and time horizon, if applicable.	Yes
Effect of engagement with patients and others affected by the study	25	Report on any difference patient/service recipient, general public, community, or stakeholder involvement made to the approach or findings of the study	Not applicable
**Discussion**			
Study findings, limitations, generalisability, and current knowledge	26	Report key findings, limitations, ethical or equity considerations not captured, and how these could affect patients, policy, or practice.	Yes
Other relevant information			
Source of funding	27	Describe how the study was funded and any role of the funder in the identification, design, conduct, and reporting of the analysis	Yes
Conflicts of interest	28	Report authors conflicts of interest according to journal or International Committee of Medical Journal Editors requirements.	Yes

From: Husereau, D., Drummond, M., Augustovski, F., de Bekker-Grob, E., Briggs, A. H., Carswell, C., et al. (2022). Consolidated health economic evaluation reporting standards 2022 (CHEERS 2022) statement: Updated reporting guidance for health economic evaluations. *MDM Policy Pract.* 7(1), 23814683211061097. doi:10.1177/23814683211061097

Supplementary Table S2 The proportions of patients receiving subsequent anti-cancer therapy and the probabilities of adverse events listed in the Phase III EXTENTORCH clinical trial.

Parameters	Estimate	SA range	Distribution
Proportion of receiving anti-tumor treatment for toripalimab group (%)			
Irinotecan	49.30	39.44~59.16	Beta
Anlotinib	32.30	25.84~38.76	Beta
Atezolizumab	13.90	11.12~16.68	Beta
Bevacizumab	0.90	0.72~1.08	Beta
Proportion of receiving anti-tumor treatment for placebo group (%)			
Irinotecan	59.40	47.52~71.28	Beta
Anlotinib	43.80	35.04~52.56	Beta
Atezolizumab	25.10	20.08~30.12	Beta
Bevacizumab	1.40	1.12~1.68	Beta
Probabilities of AEs in toripalimab group (%)			
Decreased neutrophil count	74.30	59.44~89.16	Beta
Decreased WBC count	38.70	30.96~46.44	Beta
Anemia	30.60	24.48~36.72	Beta
Decreased platelet count	24.80	19.84~29.76	Beta
Hyponatraemia	6.30	5.04~7.56	Beta
Probabilities of AEs in placebo group (%)			
Decreased neutrophil count	75.00	60.00~90.00	Beta
Decreased WBC count	44.90	35.92~53.88	Beta
Anemia	34.70	27.76~41.64	Beta
Decreased platelet count	34.30	27.44~41.16	Beta
Hyponatraemia	6.50	5.20~7.80	Beta

Abbreviations: AE, adverse event; SA, sensitivity analysis; WBC, white blood cell.

Supplementary Table S3 Summary of the statistical goodness-of-fit of Kaplan Meier survival curves in overall population.

Model	AIC for toripalimab	BIC for toripalimab	AIC for placebo	BIC for placebo
**Overall survival**				
Exponential	1399.013	1402.42	1443.233	1446.622
Weibull	1362.69	1369.504	1390.067	1396.845
Gamma	1357.299	1364.113	1384.488	1391.266
Generalized Gamma	1358.603	1368.824	1386.249	1396.416
Gompertz	1385.465	1392.28	1415.396	1422.174
Log-Normal	1371.111	1377.925	1401.308	1408.086
Log-Logistic	1347.387	1354.201	1378.443	1385.221
**Progression-free survival**				
Exponential	1082.25	1085.657	1093.619	1097.008
Weibull	1051.083	1057.897	966.8853	973.6635
Gamma	1039.579	1046.393	962.4329	969.2111
Generalized Gamma	1032.925	1043.147	963.1259	973.2932
Gompertz	1078.511	1085.326	1013.947	1020.725
Log-normal	1032.071	1038.885	988.9819	995.76
Log-Logistic	1019.859	1026.673	957.6846	964.4628

Abbreviations: AIC, Akaike information criterion; BIC, Bayesian information criterion.

Supplementary Table S4 Summary of the statistical goodness-of-fit of Kaplan Meier survival curves in HLA-A11/B62 subgroup.

Model	AIC for toripalimab	BIC for toripalimab	AIC for placebo	BIC for placebo
**OS of HLA-A11-/B62+**				
Exponential	661.70	664.34	641.96	644.52
Weibull	647.22	652.49	615.59	620.72
Gamma	645.73	650.99	613.03	618.16
Generalized Gamma	647.69	655.59	614.91	622.61
Gompertz	656.27	661.54	627.15	632.28
Log-normal	653.61	658.88	618.47	623.60
Log-Logistic	643.00	648.27	611.34	616.46
**PFS of HLA-A11-/B62+**				
Exponential	512.44	515.08	499.82	502.38
Weibull	490.43	495.70	437.01	442.14
Gamma	486.31	491.58	433.64	438.77
Generalized Gamma	487.96	495.86	435.26	442.95
Gompertz	506.76	512.02	461.89	467.02
Log-normal	492.66	497.93	444.38	449.50
Log-Logistic	479.88	485.15	432.90	438.03
**OS of HLA-A11+/B62-**				
Exponential	289.63	291.52	344.91	346.86
Weibull	269.83	273.62	331.43	335.33
Gamma	267.34	271.12	328.97	332.87
Generalized Gamma	268.18	273.85	329.60	335.46
Gompertz	276.87	280.65	338.80	342.71
Log-normal	266.21	269.99	327.64	331.54
Log-Logistic	266.42	270.20	327.57	331.47
**PFS of HLA-A11+/B62-**				
Exponential	197.17	199.06	269.52	271.47
Weibull	190.09	193.87	243.88	247.78
Gamma	187.87	191.66	244.33	248.23
Generalized Gamma	187.73	193.41	245.71	251.56
Gompertz	195.94	199.73	250.58	254.48

Log-normal	185.80	189.58	248.42	252.32
Log-Logistic	185.38	189.16	247.56	251.46

Abbreviations: AIC, Akaike information criterion; BIC, Bayesian information criterion; OS, overall survival; PFS, progression-free survival; HLA, human leukocyte antigen.

Supplementary Table S5 Summary of the statistical goodness-of-fit of Kaplan Meier survival curves in ITH subgroup.

Model	AIC for toripalimab	BIC for toripalimab	AIC for placebo	BIC for placebo
**OS of ITH high**				
Exponential	584.25	586.68	420.48	422.62
Weibull	547.73	552.59	400.43	404.72
Gamma	548.47	553.34	401.79	406.08
Generalized Gamma	549.51	556.80	402.43	408.86
Gompertz	555.86	560.72	405.18	409.46
Log-normal	557.80	562.66	413.80	418.09
Log-Logistic	551.17	556.03	404.03	408.32
**PFS of ITH high**				
Exponential	415.38	417.81	335.63	337.77
Weibull	358.39	363.26	292.03	296.32
Gamma	360.54	365.40	295.41	299.69
Generalized Gamma	360.17	367.46	293.99	300.42
Gompertz	370.59	375.45	303.30	307.59
Log-normal	369.45	374.31	307.23	311.52
Log-Logistic	362.50	367.36	299.70	303.98
**OS of ITH low**				
Exponential	357.39	359.61	508.61	511.05
Weibull	356.78	361.22	492.82	497.71
Gamma	356.62	361.06	487.51	492.39
Generalized Gamma	358.62	365.28	480.02	487.35
Gompertz	358.71	363.15	504.47	509.35
Log-normal	362.39	366.83	480.44	485.33
Log-Logistic	355.27	359.71	482.04	486.92
**PFS of ITH low**				
Exponential	298.79	301.01	371.44	373.88
Weibull	297.90	302.34	327.61	332.50
Gamma	296.46	300.90	323.54	328.43
Generalized Gamma	296.19	302.85	325.39	332.72
Gompertz	300.78	305.22	345.48	350.36

Log-normal	294.63	299.06	324.76	329.65
Log-Logistic	291.73	296.17	323.81	328.69

Abbreviations: AIC, Akaike information criterion; BIC, Bayesian information criterion; OS, overall survival; PFS, progression-free survival; HLA, human leukocyte antigen.

Supplementary Table S6 Optimal distribution and survival parameters for all subgroup.

Group	Model	Parameters
**A11-/B62+ subgroup**		
OS of toripalimab	Log-logistic	scale=13.263; shape=2.13
OS of placebo	Log-logistic	scale=13.512; shape=2.484
PFS of toripalimab	Log-logistic	scale=5.546; shape=2.511
PFS of placebo	Log-logistic	scale=5.232; shape=3.496
**A11+/B62- subgroup**		
OS of toripalimab	Log-normal	meanlog=3.0096; sdlog=0.5632
OS of placebo	Log-logistic	scale=13.94; shape=2.54
PFS of toripalimab	Log-logistic	scale=7.813; shape=2.369
PFS of placebo	Log-logistic	scale=5.169; shape=2.898
**ITH high subgroup**		
OS of toripalimab	Weibull	scale=16.485; shape=1.929
OS of placebo	Weibull	scale=17.35; shape=1.78
PFS of toripalimab	Weibull	scale=6.575; shape=2.344
PFS of placebo	Weibull	scale=6.068; shape=2.218
**ITH low subgroup**		
OS of toripalimab	Log-logistic	scale=21.021; shape=1.607
OS of placebo	Log-normal	meanlog=2.639; sdlog=0.7092
PFS of toripalimab	Log-logistic	scale=7.548; shape=1.768
PFS of placebo	Gamma	shape=4.073; rate=0.657

Abbreviations: OS, overall survival; PFS, progression-free survival; ITH, intra-tumor heterogeneity.

Supplementary Table S7 Summary of INMB in base-case and subgroup analysis at WTP thresholds of 1-3 times per capita GDP.

Group	WTP=$13,445/QALY	WTP=$26,889/QALY	WTP=$40,334/QALY
Base-case	-1,273.75[a]	1,324.88	3,923.70
	-1,230.50[b]	1,368.13	3,966.94
A11+B62-	-1,527.53	2,814.39	7,156.64
	-1,509.73	2,832.19	7,174.43
A11-B62+	-507.28	532.04	1,571.44
	-412.84	626.48	1,665.88
ITH high	-91.43	-193.33	-295.24
	-101.91	-203.81	-305.72
ITH low	-3,520.57	5,388.29	14,297.82
	-3,660.91	5,247.95	14,157.48

Abbreviations: INMB, incremental net monetary benefit; WTP, willingness-to-pay; GDP, gross domestic product; QALY, quality-adjusted life year; ITH, intra-tumor heterogeneity; HLA, human leukocyte antigen.

a: Without drug wastage; b: With drug wastage.

Supplementary Table S8 Summary of NMB in base-case and subgroup analysis at WTP thresholds of 1-3 times per capita GDP.

Group	WTP=$13,445/QALY	WTP=$26,889/QALY	WTP=$40,334/QALY
**Base-case**			
Toripalimab + chemotherapy	-18,523.83[a]	-8,497.04	1,530.51
	-19,039.57[b]	-9,012.77	1,014.77
Placebo + chemotherapy	-17,250.08	-9,821.91	-2,393.19
	-17,809.07	-10,380.90	-2,952.18
**A11+B62-**			
Toripalimab + chemotherapy	-20,051.35	-7,860.60	4,331.06
	-20,613.94	-8,423.19	3,768.47
Placebo + chemotherapy	-18,523.82	-10,674.99	-2,825.58
	-19,104.21	-11,255.38	-3,405.97
**A11-B62+**			
Toripalimab + chemotherapy	-19,151.13	-10,514.23	-1,876.70
	-19,638.83	-11,001.94	-2,364.40
Placebo + chemotherapy	-18,643.85	-11,046.28	-3,448.14
	-19,225.99	-11,628.42	-4,030.28
**ITH high**			
Toripalimab + chemotherapy	-18,266.83	-12,002.78	-5,738.28
	-18,685.37	-12,421.33	-6,156.82
Placebo + chemotherapy	-18,175.40	-11,809.45	-5,443.04
	-18,583.46	-12,217.52	-5,851.10
**ITH low**			
Toripalimab + chemotherapy	-22,223.85	-5,417.47	11,390.17
	-22,956.93	-6,150.54	10,657.10
Placebo + chemotherapy	-18,703.29	-10,805.76	-2,907.64
	-19,296.02	-11,398.49	-3,500.37

Abbreviations: NMB, net monetary benefit; WTP, willingness-to-pay; GDP, gross domestic product; QALY, quality-adjusted life year; ITH, intra-tumor heterogeneity.

a: Without drug wastage; b: With drug wastage.

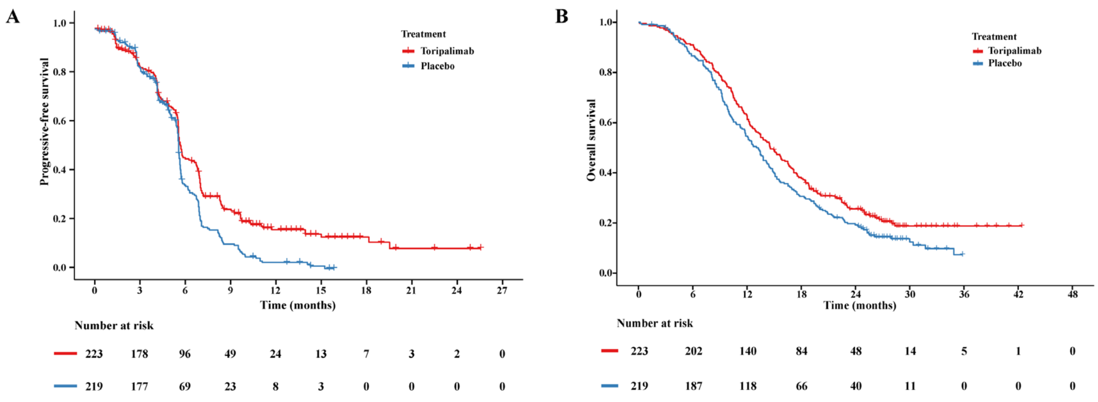

Supplementary Figure S1 Reconstruction of Kaplan Meier survival curve in the intention-to-treat population. (A) PFS curve. (B) OS curve.

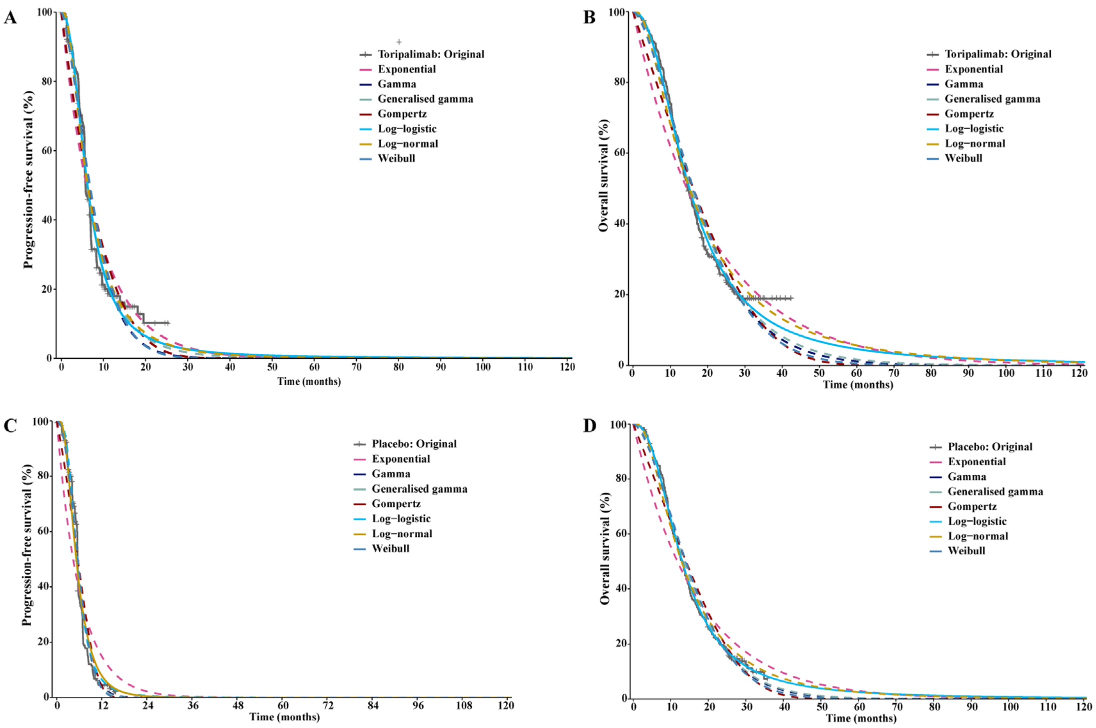

Supplementary Figure S2 Fitting and extrapolation of Kaplan Meier survival curve in the intention-to-treat population. (A)Toripalimab plus chemotherapy PFS curve. (B)Toripalimab plus chemotherapy OS curve. (C) Placebo plus chemotherapy PFS curve. (D) Placebo plus chemotherapy OS curve.

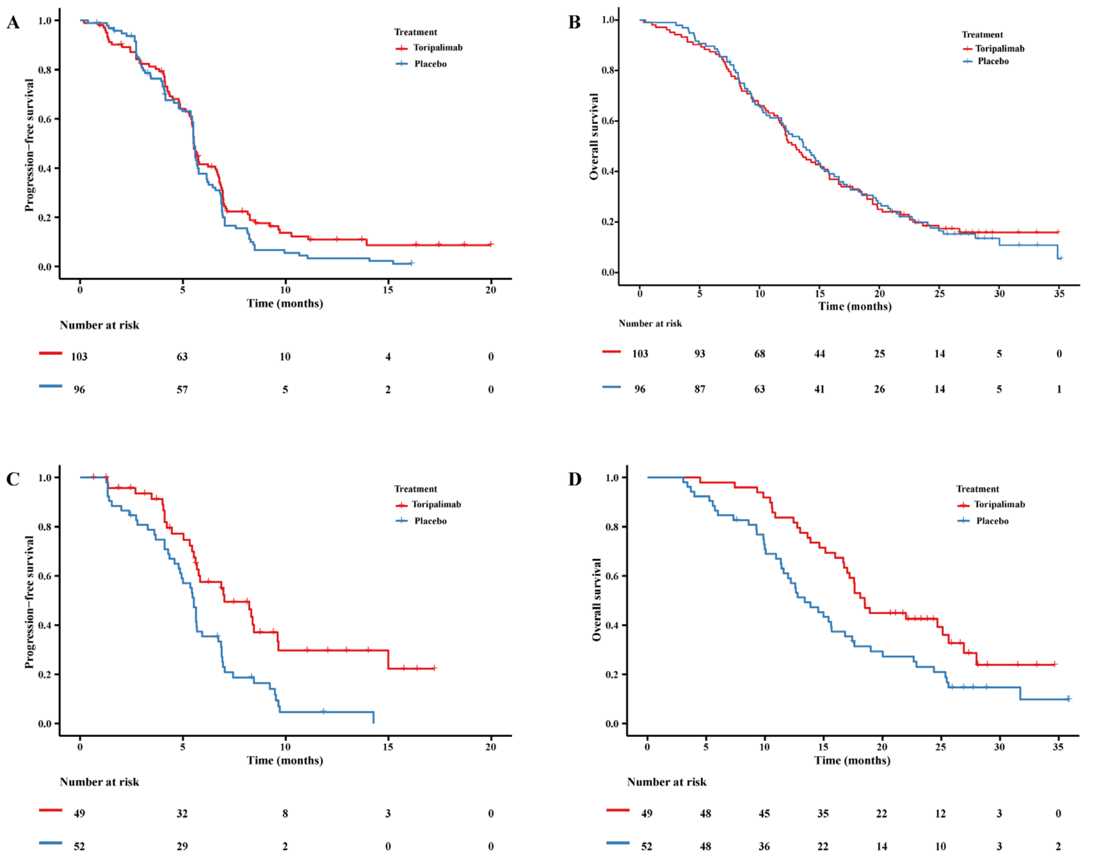

Supplementary Figure S3 Reconstruction of Kaplan Meier survival curve in HLA-A11/B62 subgroup. (A) PFS curve in A11$^-$/B62$^+$ group. (B) OS curve in in A11$^-$/B62$^+$ group. (C) PFS curve in A11$^+$/B62$^-$ group. (D) OS curve in A11$^+$/B62$^-$ group.

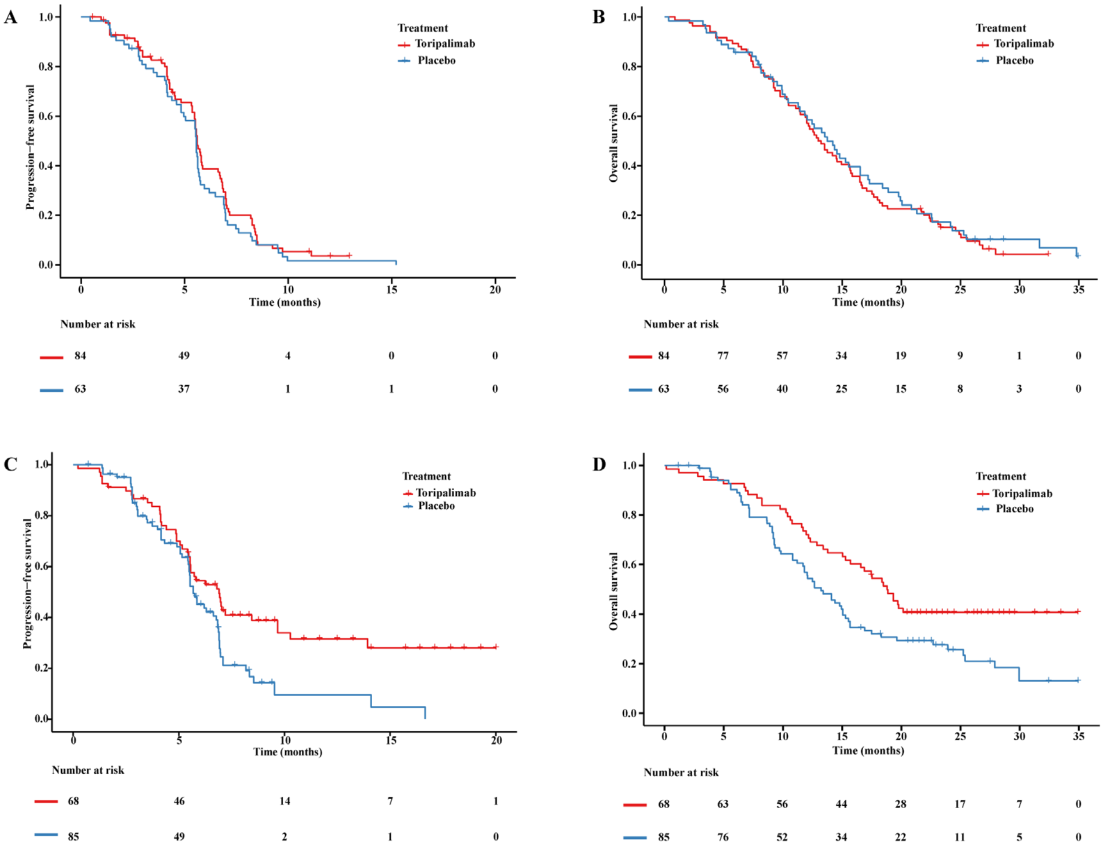

Supplementary Figure S4 Reconstruction of Kaplan Meier survival curve in ITH subgroup. (A) PFS curve in ITH high group. (B) OS curve in in ITH high group. (C) PFS curve in ITH low group. (D) OS curve in ITH low group.

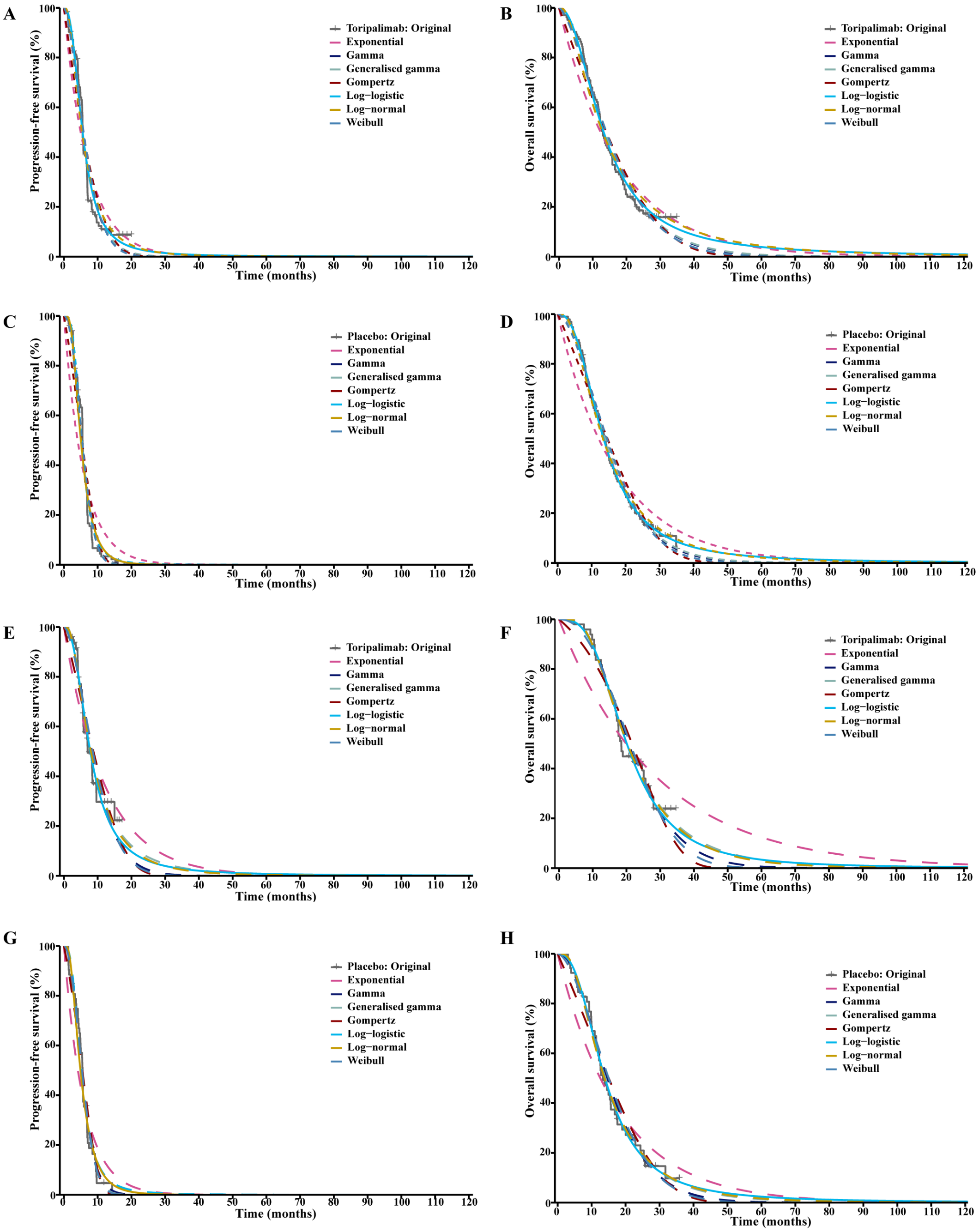

Supplementary Figure S5 Fitting and extrapolation of Kaplan Meier survival curve in HLA-A11/B62 subgroup. (A)Toripalimab plus chemotherapy PFS curve in A11$^-$/B62$^+$ group. (B) Toripalimab plus chemotherapy OS curve in A11$^-$/B62$^+$ group. (C) Placebo plus chemotherapy PFS curve in A11$^-$/B62$^+$ group. (D) Placebo plus chemotherapy OS curve in A11$^-$/B62$^+$ group. (E)Toripalimab plus chemotherapy PFS curve in A11$^+$/B62$^-$ group. (F) Toripalimab plus chemotherapy OS curve in A11$^+$/B62$^-$ group. (G) Placebo plus chemotherapy PFS curve in A11$^+$/B62$^-$ group. (H) Placebo plus chemotherapy OS curve in A11$^+$/B62$^-$ group.

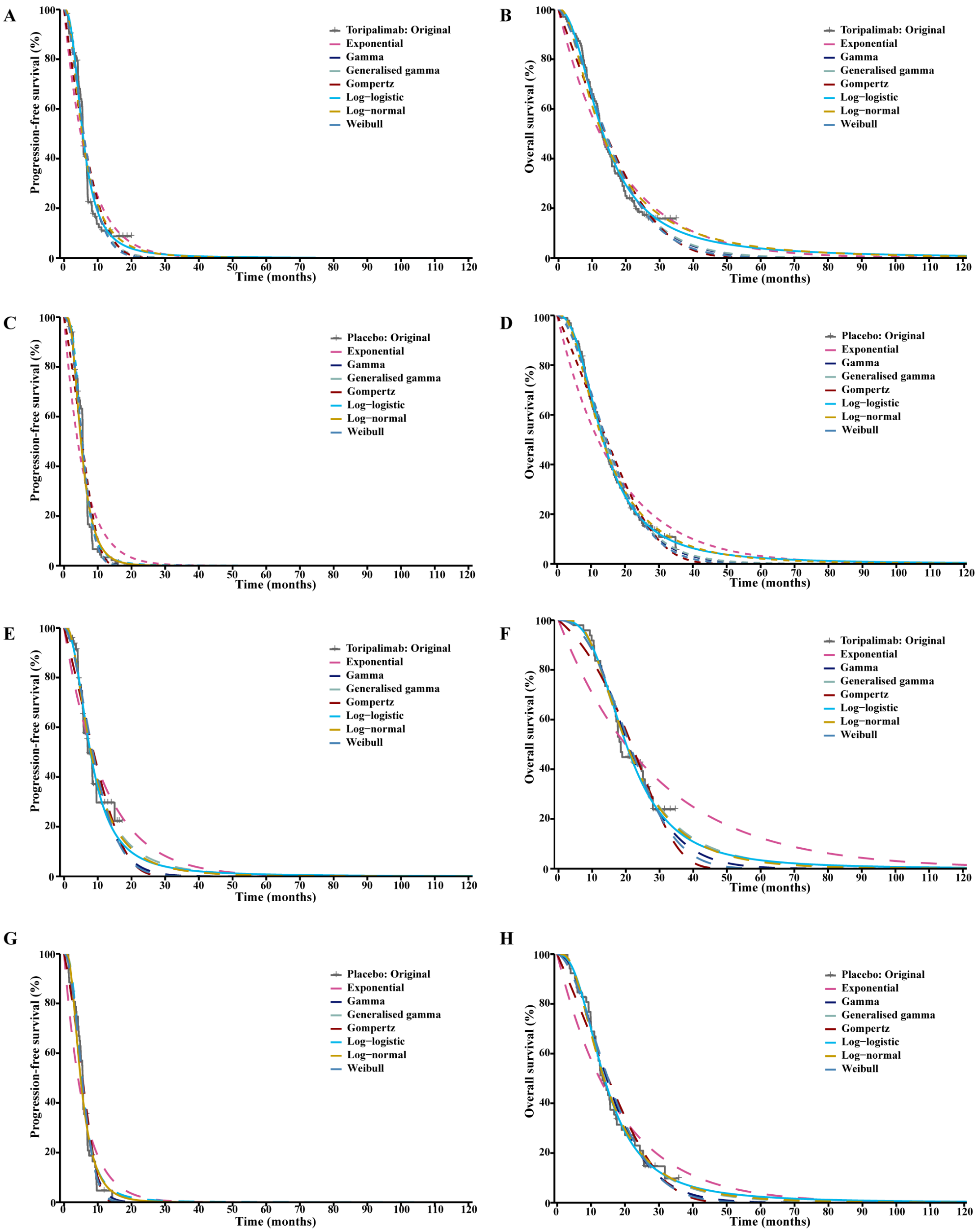

Supplementary Figure S6 Fitting and extrapolation of Kaplan Meier survival curve in ITH subgroup. (A)Toripalimab plus chemotherapy PFS curve in ITH high group. (B) Toripalimab plus chemotherapy OS curve in ITH high group. (C) Placebo plus chemotherapy PFS curve in ITH high group. (D) Placebo plus chemotherapy OS curve in ITH high group. (E)Toripalimab plus chemotherapy PFS curve in ITH low group. (F) Toripalimab plus chemotherapy OS curve in ITH low group. (G) Placebo plus chemotherapy PFS curve in ITH low group. (H) Placebo plus chemotherapy OS curve in ITH low group.
